# Mutagenesis of N-terminal residues confer thermostability on a *Penicillium janthinellum* MA21601 xylanase

**DOI:** 10.1186/s12896-019-0541-7

**Published:** 2019-07-25

**Authors:** Ke Xiong, Jie Hou, Yuefeng Jiang, Xiuting Li, Chao Teng, Qin Li, Guangsen Fan, Ran Yang, Chengnan Zhang

**Affiliations:** 1 Beijing Advanced Innovation Center for Food Nutrition and Human Health, Beijing Technology and Business University, No 11 Fucheng Street, Haidian District, Beijing, 100084 China; 2 Beijing Engineering and Technology Research Center of Food Additives, Beijing Technology and Business University, No 11 Fucheng Street, Haidian District, Beijing, 100084 China; 3 Beijing Key Laboratory of Flavor Chemistry, Beijing Technology and Business University, No 11 Fucheng Street, Haidian District, Beijing, 100084 China; 4 School of Food and Chemical Engineering, Beijing Technology and Business University, Beijing, 100048 China

**Keywords:** Endo-1, 4-β-xylanase, Thermostability, N-terminal region, Disulfide bridge, Enzyme mutation

## Abstract

**Background:**

A mesophilic xylanase PjxA from *Penicillium janthinellum* MA21601 has high specific activity under acidic condition and holds great potential for applications in the animal feed industry. To enhance the thermostability of xylanase PjxA, two mutation strategies in the N-terminal region were examined and then integrated into the xylanase to further improvement. The recombinant xylanase PTxA-DB (The meaning of DB is disulfide-bridge.) was constructed by replacement of five residues in the mutated region in TfxA (T10Y, N11H, N12D, Y15F, N30 L), combined with an additional disulfide bridge in the N-terminal region.

**Results:**

The *T*_*m*_ value of mutant PTxA-DB was improved from 21.3 °C to 76.6 °C, and its half-life was found to be 53.6 min at 60 °C, 107-fold higher than the wild type strain. The location of the disulfide bridge (T2C-T29C) was between the irregular loop and the β-strand A2, accounting for most of the improvement in thermostability of PjxA. Further analysis indicated T2C, T29C, N30 L and Y15F lead to increase N-terminal hydrophobicity. Moreover, the specific activity and substrate affinity of PTxA-DB were also enhanced under the acidic pH values.

**Conclusions:**

These results indicated PTxA-DB could be a prospective additive to industrial animal feeds.

**Electronic supplementary material:**

The online version of this article (10.1186/s12896-019-0541-7) contains supplementary material, which is available to authorized users.

## Background

Endo-1,4-β-xylanases (EC 3.2.1.8) are glycoside hydrolases that catalyze the cleavage of internal *β*-1, 4-D-xylosidic linkages of xylan the major constituent of hemicellulose [1]. They have attracted much attention in recent years due to their biotechnological applications, including in the pulp and paper, food and feed industries [2]. To meet the different performance needs for xylanases, many efforts have been made to improve their properties. It is known that a wide variety of microorganisms can produce xylanases, but many microbial xylanases lack the physicochemical properties required of industrial applications [3], such as being functional under acidic conditions and high temperatures. Most of the acidic xylanases were isolated from fungi, rather than bacteria. Furthermore, acidic xylanases have fewer salt bridges than alkaline xylanases, therefore have lower thermostability [4], which makes them unsuitable for industrial processes, such as animal feed and baking, where processing is mostly done at high temperatures. Moreover, the use of some thermostable xylanases in industrial applications is restricted further by their poor activities or low stability at extreme pH [5–7].

Xylanases are produced by diverse variety of micro-organisms including extremophilic fungi, bacteria, yeast etc. which are classified in several glycoside hydrolase (GH) families (5, 7, 8, 10, 11, 26, 30 and 43). Research has mainly focused on only two of the xylanase containing glycoside hydrolase families, namely families 10 and 11, yet enzymes with xylanase activity belonging to families 5, 7, 8, 26,30 and 43 have also been identified and studied, albeit to a lesser extent. [8, 9]. Glycoside hydrolases family 11 (GH11) xylanase resembles the shape of a partially closed right hand and consists of one α-helix and two twisted antiparallel β-sheets. In terms of industrial applications, GH11 xylanases are attractive due to their small molecular mass, high substrate selectivity, and variety of pH optima [10, 11]. Recently, there have been extensive studies on the structural features of GH11 xylanase to understand the mechanisms of their thermostability. It has been found various kinds of interactions contributed to enzyme thermostability, including hydrogen bonding, salt and disulfide bridges, and hydrophobic forces [12]. Notably, many researchers have suggested that the N-terminus of the enzyme plays a critical role in the stability of GH11 xylanases since unfolding is initiated in this region [13]. Therefore, several groups have attempted to improve the thermostability of xylanases by focusing on the N-terminal region. Replacement of the entire N-terminus of mesophilic xylanases or site-directed mutations of the corresponding region from thermophilic xylanases has given increased heat resistance [14, 15]. *Thermomonospora fusca* TfxA is known as one of the most thermostable wild type xylanases [16], *Streptomyces olivaceovirdis* SoxB has achieved a significant increase in thermostability of mesophilic xylanase by substituting the corresponding N-terminal region of TfxA. Furthermore, the N-terminal of TfxA was divided into four regions (Region 1, Region2, Region3 and Region4) to produce four regional mutants, it has been reported that only five mutations in region 2 might provide critical effect, rather than the strategies for TfxA replacing N-terminal residues involved in conferring thermostability. [16, 17].

Besides, N-terminal disulfide bridges have been engineered to increase the thermostability of several GH11 xylanases [18–20]. EvXyn11^TS^ is an example of a successfully engineered hyperthermostable GH11 xylanase, which has a disulfide bridge in the N-terminal region [21]. A hybrid xylanase AEXynM with high thermostability was constructed by substituting N-terminal 33 amino acids of AuXyn11A with corresponding 38 amino acids of EvXyn11^TS^. These results further verified that Cys5-Cys32 in the newly constructed N-terminus of the enzyme contributes mainly to the high thermostability of AEXynM [15]. Yin et al. used the EvXyn11^TS^ gene sequence encoding a thermophilic xylanase SyXyn11, which contains a disulfide bridge (Cys5-Cys32) in the N-terminus. Then, by mutating the Cys5 amino acid residue of Syxyn11 to Thr5, the temperature optimum and stability of reSyXyn11 of 85 °C and 80 °C decreased to 70 °C and 50 °C, respectively, which indicated there is a close correlation between the N-terminal disulfide bridge and thermostability [22].

In this study, an acidic xylanase *pjxA* gene was cloned from *Penicillium janthinellum* (MA21601) and continually expressed in *Escherichia coli* BL21 (DE3). The expressed recombinant xylanase PjxA showed high specific activity and stability under acidic conditions. However, the thermostability of PjxA must be enhanced further if it is to be used in current industrial processes. For example, a short time, high-temperature treatment is necessary for the animal feed process, so the xylanases must be stable at elevated temperature and acidic pH values if used as a feed additive. Therefore, the industrial application of PjxA was prevented by its weak heat-resistance. Authors from previous studies have postulated that the higher stability of thermophilic GH11 xylanases may be dependent on different features that may have contributed to their increase in thermostability. A variety of protein engineering strategies also have been found to have different effects. Based on the heat resistance mechanism in the GH11 natural thermostable enzyme TfxA and the engineering hyperthermostable enzyme EvXyn11^TS^, xylanase PTxA was created by substituting five residues, four of them (T10Y, N11H, N12D, Y15F) in the N-terminal region of TfxA and the remaining mutation site, N30 L, modified to the sequence consistent with the corresponding site of the enzyme TfxA and EvXyn11^TS^. This mutation site may have the synergism with N-terminal mutated region. The engineered PjxA-DB xylanase has introduced an extra disulfide bridge in the corresponding position of EvXyn11^TS^ N-terminal region. The reason for PjxA being unstable at high temperatures will be analyzed by the impact of these two mutation strategies and further developed to incorporate both mutation methods to further improve the thermostability.

## Results

### Recombinant xylanases design

**Fig. 1:**
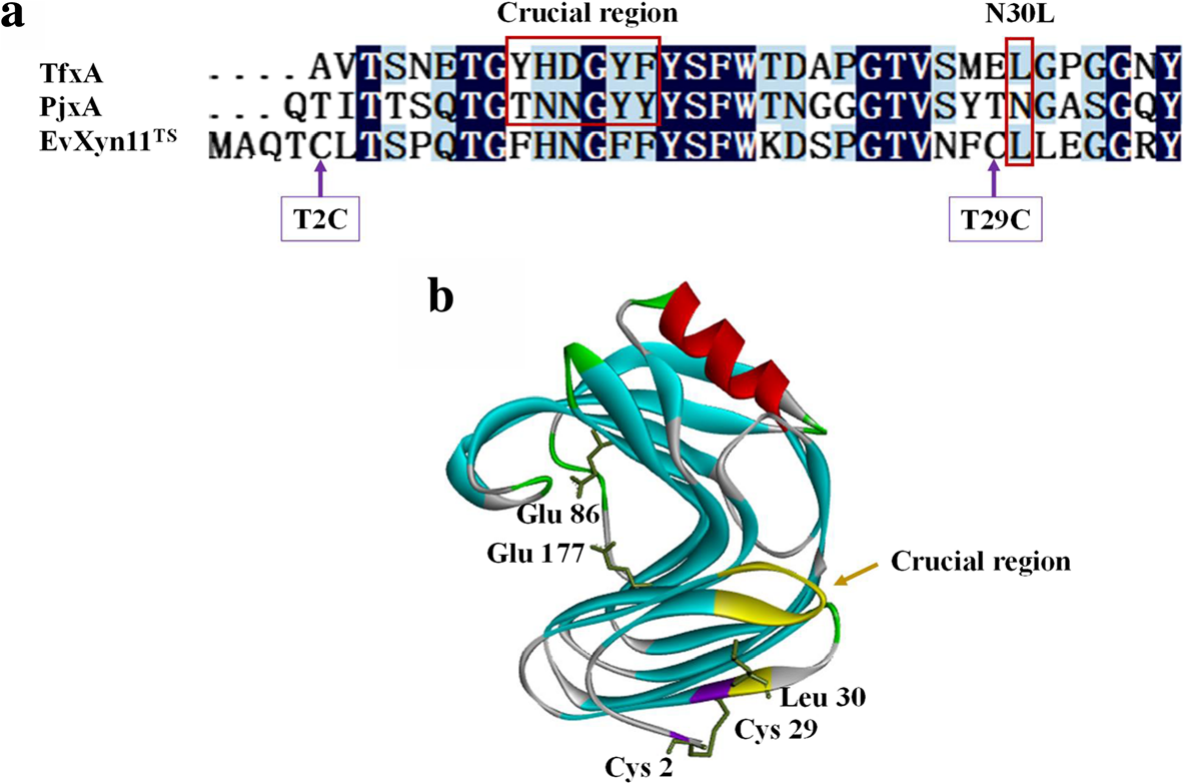
(**a**) Amino acid sequence comparison of the N-terminal sequence of PjxA and other xylanases. DNAMAN 6.0 software and NCBI (https://www.ncbi.nlm.nih.gov/) were used to compare the amino acid sequence of acid xylanase PjxA and other xylanases. PjxA: Recombinant xylanase of *Penicillium janthinellum* (MA21601); TfxA: Thermostable xylanase from *Thermomonospora fusca*; EvXyn11^TS^: Engineering hyperthermostable enzyme. (**b**) The structure of the mutant PTxA-DB based on PDB: 3wp3.1A, an additional disulfide bridge between Cys2-Cys29 displayed in purple; mutations of Leu30 and mutated region indicated in yellow. The prediction and homology modeling of the 3D structure of PjxA were performed by SWISS-MODEL (http://swissmodel.expasy.org/)

The cDNAs constructs coding for PjxA, PTxA, PjxA-DB and PTxA-DB were inserted into the expression vector PET-28a and confirmed by sequencing. PTxA substituted its N-terminal residues (T10Y, N11H, N12D, Y15F) with the mutated region from TfxA [16], which has been reported to account for much of the improvement in thermostability of the N-terminal mutants. Meanwhile, L30 contributed to thermostability showing synergism with this region, and the Leu residue was also found in the corresponding position of the hyperthermostable xylanase EvXyn11^TS^. Sequence alignment has indicated that T2C and T29C in xylanase PjxA-DB might improve the thermostability by introducing an additional disulfide bridge (Fig. 1a). These two recombinant xylanases, derived from the two mutation methods, were further combined to generate a PTxA-DB, which contained a disulfide bridge and additional mutated sites of five residues (Fig. 1b).

### Expression and purification of PjxA and mutants

**Fig. 2:**
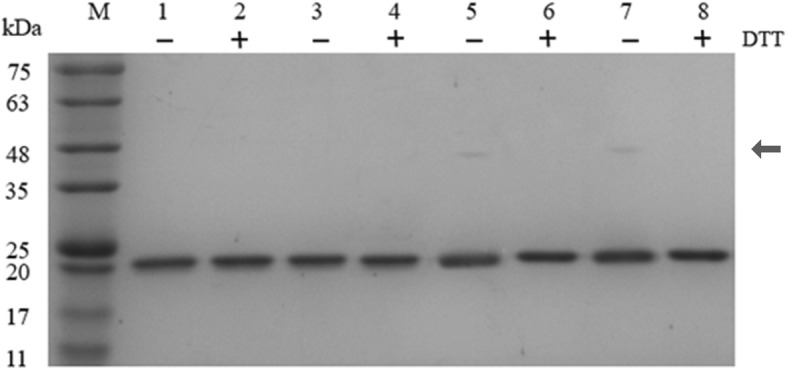
SDS-PAGE analysis of the purified xylanases. Samples were treated in 1% SDS and the presence or absence of 2 mM DTT at 70 °C for 5 min. Lane M, the molecular weight marker; lane 1, PjxA(−DTT); lane 2, PjxA(+DTT); lane 3, PTxA(−DTT); lane 4, PTxA(+DTT); lane 5, PjxA-DB(−DTT); lane 6, PjxA-DB(+DTT); lane 7, PTxA-DB(−DTT); lane 8, PTxA-DB(+DTT). The arrowhead indicates the dimer of PjxA-DB and PTxA-DB

The recombinant xylanases were successfully expressed in *E. coli* BL21 (DE3) and checked by SDS-PAGE analysis after Ni-column purification. Because of the wild type, similar to other recombinant types by having a free Cys residue on the protein surface (C43), we added the reducing agent to detect intermolecular disulfide bridges. Four recombinant proteins were incubated with 1% SDS in the absence and presence of DTT (2 mM) at 70 °C for 5 min. As shown in Fig. 2(Additional file 1: Figure S1), PjxA-DB and PTxA-DB formed a few dimers in the absence of reducing agent in lanes 5 and 7. The monomer and dimer of those two mutated recombinant xylanases were separately analyzed through LC-MS, and the results indicated the formation of intramolecular disulfide bridge C2–29 in the monomer, and a small amount of unspecific intermolecular disulfide bridge in the dimer (Additional file 1: Figure S2-S5).

### Optimal pH and pH stability of PjxA and mutants

**Fig. 3:**
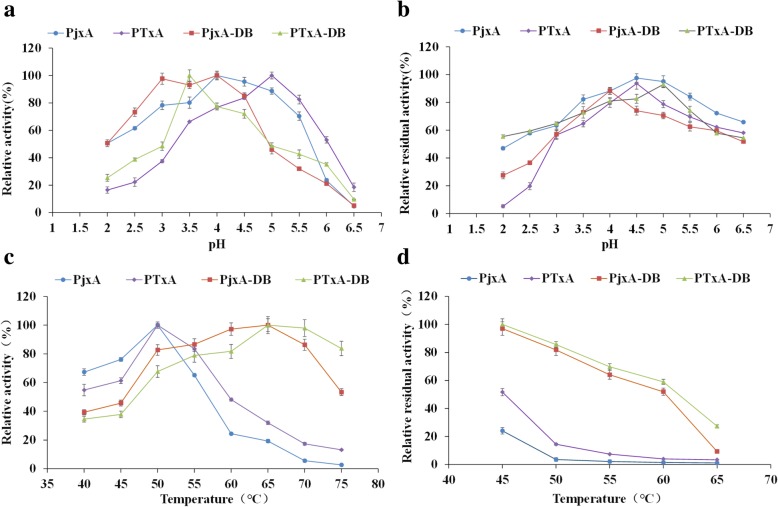
(**a**) Optimal pH and (**b**) pH stability of PjxA and the three mutants. For pH stability, all proteins were pre-incubated at 40 °C for 30 min before measuring enzymatic activity. The amount of the enzyme was adjusted to 0.05 μg in each reaction. The sample controls without pre-incubation were defined as 100%. (**c**) Optimal temperature and (**d**) thermostability of PjxA and the three mutants. For thermal stability, all xylanases were incubated at 50 mM pH 5.5 sodium acetate buffer for 30 min before activity assay. The amount of the enzyme was adjusted to 0.05 μg in each reaction. The samples measures without pre-incubation were defined as 100%. Results are presented as means ± SD of duplicate experiments

The optimal pH was investigated with 10 mg/mL beechwood xylan for 5 min at a pH range of 2.0–6.5. The optimal pH for the PjxA is 4.0 (Fig. 3a), for both PTxA-DB and PjxA-DB are pH 3.5–4.0, while for PTxA is pH 5.0, slightly higher than PjxA. All mutants were incubated at 40 °C for 30 min without a substrate to measure the pH stability of the recombinant xylanases. At pH 2.0, the residual activities was 55% for PTxA-DB, more than 10% for PjxA, 27% for PjxA-DB, and 5% for PTxA (Fig. 3b). After incubation between pH 3.5 to 5.5, for 30 min at 40 °C, all recombinant xylanases tested were stable and showed over 60% residual activity. The highest residual activity was found at pH 4.5 for PjxA and PTxA at 97 and 93%, respectively. Besides, the residual activities of the recombinant xylanases were similar between pH 6.0–6.5.

### Temperature characteristics of PjxA and mutated recombinant xylanases

The temperature optimum was determined by measuring residual activity on 10 mg/mL beech-wood xylan for 5 min at different temperatures (Fig. 3c), the temperature optimum for both PjxA and PTxA are 50 °C, yet the optimal temperature increased to 65 °C for PjxA-DB and PTxA-DB, indicating that PjxA acquired a thermophilic property after introducing an extra disulfide bond in the N-terminal region. Furthermore, PTxA-DB exhibited over 80% relative activity at 75 °C. The four recombinant xylanases were tested for residual activity after incubation in the temperature range of 45–65 °C for 30 min to determine their respective thermostabilities. As shown in Fig. 3(d), PjxA had only 4% residual activity after incubating 30 min at 50 °C, and was inactivated when the temperature increased to 55 °C. PTxA showed slightly higher residual activities of 15 and 7% at 50 °C and 55 °C, respectively. However, PjxA-DB and PTxA-DB retained 52 and 60% residual activity at 60 °C, this being significantly higher than PjxA. The disulfide bridge in PjxA-DB and PTxA-DB were removed by DTT to check the importance of having a disulfide bridge to enhance heat resistance and the result showed that they had lower activities compared to not being treated with DTT. The relative residual activity of PjxA-DB declined to 5%, PTxA-DB declined to 12%, for wild type PjxA and recombinant PTxA with DTT remained unchanged (Additional file 1: Table S1).

To compare the thermostability of the mutated xylanases, the half-lives of inactivation (*t*_1/2_) were determined at 60 °C (Table 1). The half-life of PTxA was found to be 58 s, approximately 2-fold higher than that of PjxA (30 s), while PjxA-DB and PTxA-DB had much higher thermostability with half-lives of 33.8 min and 53.6 min. The later results represent an impressive 68-fold and 107-fold increase over that of PixA.

**Table 1:** The t_1/2_ at 60 °C and midpoint of thermal unfolding of recombinant xylanases

Xylanases	Mutations	*t*_1/2_ at 60 °C	*T*m (^o^C)	Δ*T*m(^o^C)
PjxA	–	30 s	55.3 ± 0.4	–
PTxA	T10Y/N11H/N12D/Y15F/N30 L	58 s	58.4 ± 0.5	3.1 ± 0.9
PjxA-DB	T2C/T29C	33.8 min	72.7 ± 0.6	17.4 ± 1.0
PTxA-DB	T2C/T10Y/N11H/N12D/Y15F/N30 L/T29C	53.6 min	76.6 ± 0.6	21.3 ± 1.0

The half-lives at 60 °C were determined as residual activity by incubating at different times at 60 °C. All protein concentrations were 0.05 mg/mL, and the midpoint of thermal unfolding (*T*_m_) was determined by differential scanning calorimetry. The parallel experiments in 3 times

**Fig. 4:**
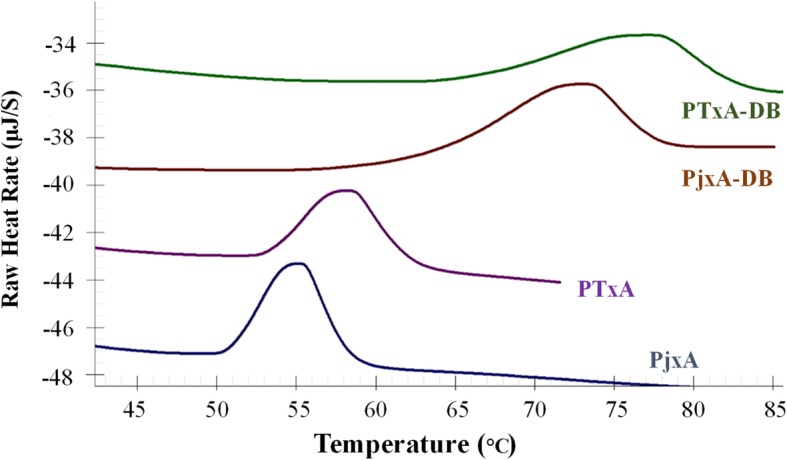
DSC results of PjxA (blue line), PTxA (purple line), PjxA-DB (red line) and PTxA-DB (green line). All samples were adjusted to 0.5 mg/mL and dialyzed against 50 mM sodium acetate buffer (pH 5.5) before measuring by DSC. A graph was generated by the Launch Nano Analyze software

The *T*_m_ of mutated recombinant xylanases were investigated by differential scanning calorimetry to further validate the improved thermostability in the recombinant enzymes (Table 1 and Fig. 4). The *T*_*m*_ values of PTxA and PjxA-DB were estimated to be 58.4 °C and 72.7 °C, respectively, 3.1 °C and 17.4 °C higher than PjxA (55.3 °C). Furthermore, when the two mutation strategies were combined to generate PTxA-DB, it displayed a higher *T*_m_ value (76.6 °C) compare to the single mutation strategy.

### The kinetic parameters of PjxA and recombinant xylanases

The specific activity of all mutated recombinant xylanases increased; namely, PjxA, PTxA, PjxA-DB and PTxA-DB were 862.60 U/mg, 977.31 U/mg, 1100.27 U/mg and 1480.98 U/mg, respectively. The kinetic parameters were investigated with beechwood xylan as substrate (Table 2, Additional file 1: Figure S6). Among the mutants, PTxA-DB had the highest *kcat* value, which was 2.44-fold more than that of PTxA. The *kcat/Km* of PTxA-DB was the highest among the mutants at 85.7 mg-1·s-1 mL. Along with thermostability, PjxA-DB and PTxA-DB showed higher *kcat* values than the PjxA and PTxA. This indicates the better rate of enzymatic conversion of the mutants with the substrate. Moreover, PjxA-DB and PTxA-DB showed higher *kcat/Km* values than the PjxA and PTxA. Therefore PjxA-DB and PTxA-DB displayed higher catalytic efficiency compare to PjxA.

**Table 2:** Kinetic parameters of PjxA and its three mutated recombinant xylanases

Xylanases	*k*_cat_ (1/s)	*K*_m_ (mg/mL)	*k*_cat_/*K*_m_ (mL/s·mg)
PjxA	589.5 ± 12.3	12.6 ± 0.6	46.8
PTxA	623.6 ± 11.1	9.3 ± 0.7	67.0
PjxA-DB	1438.0 ± 14.5	20.3 ± 0.6	70.8
PTxA-DB	1191.0 ± 16.5	13.9 ± 0.8	85.7

The substrate used to measure xylanase activity was beechwood xylan. The substrate concentrations were 0.2 to 1.5% for PjxA, 0.2 to 1.0% for PTxA, 0.2 to 2.5% for PjxA-DB and 0.2 to 2.0% for PTxA-DB. All samples were adjusted to 0.05 mg/mL and dialyzed against their optimal pH, the enzyme assay was under their optimal temperatures

## Discussion

Thermostability is a desirable property of xylanases for industrial applications [23], especially for the mesophilic xylanases like PjxA which have high specific activity under acidic condition. Enhancing thermal stability is the usual requisite for enzymes to be successfully applied in the animal feed industry [24]. Previous studies have proposed that the heat-mediated unfolding of GH11 xylanases initiates from the N-terminal region [21, 25]. Therefore, mutations at the N-terminal region have been reported as a useful strategy to improve the thermostability of GH11 xylanases [26, 27]. In this study, acidic xylanase PjxA was re-engineered by replacing part of the N-terminal region with amino acid N30 L combined with introducing an extra disulfide bridge to further enhance the thermostability.

In previous studies, a significant enhancement in thermostability of mesophilic xylanase SoxB had been achieved by replacing the N-terminal region of TfxA. It has been assumed that both additive (the thermostabilizing effect of the individual mutations T11Y, N12H and Y16F) and cooperative (H12-D13 electrostatic interaction and Y11-F16 hydrophobic interaction) effects were responsible for the improvement in thermostability on SoxB [17]. In this study, we constructed recombinant PTxA by substituting four residues (T10Y, N11H, N12D and Y15F) similar to that from TfxA N-terminal region, and combining this with N30 L to produce stronger hydrophobic interaction. Additional recombinant enzymes were constructed by introducing four residues and a leucine residue separately, but the thermostability of these xylanases was only slightly improved (Additional file 1: Table S2). Since the corresponding position on the three-dimensional structure is leucine for the thermostable xylanase TfxA, as well as hyperthermostable xylanase EvXyn11^TS^, the interactions between L30 and H11 might have a synergistic effect on thermostability. However, the improvement in thermostability for PTxA was not as we anticipated and only gave a *T*_m_ value of 3.1 °C higher than PjxA. The residual activity of PTxA was over 50% after incubation for 30 min at 45 °C, but only 7% at 55 °C, suggesting that the PTxA was unstable at elevated temperature. Since thermophilic xylanases have, on average, slightly more hydrogen bonds than mesophilic xylanases [28], it is possible that D12 has a negative effect on thermostability by removing two hydrogen bonds (N12-G31 and N11-N12), although the N12D introduced electrostatic interaction with N11H, and thus might be the reason why mutant PTxA did not show marked improvement in thermostability.

However, when a disulfide bridge was constructed in the N-terminal region on PjxA, the heat resistance was significantly enhanced. The *T*_m_ value of recombinant PjxA-DB increased 17.4 °C to 72.7 °C, its half-life increased from ~ 30 s to ~ 33.8 min at 60 °C, and the optimal temperature at pH 4.0 was 15 °C higher than the wild type enzyme. Disulfide bridges are believed to stabilize proteins mostly through an entropic effect by decreasing the entropy of the protein’s unfolded state [29]. An additional N-terminal disulfide bridge (Q1C-Q24C) was introduced into *Thermomyces lanuginosus* xylanase TLX, which increased the melting temperature from 66 °C to 74 °C, and the temperature optimum shifted upwards at pH 6.5 by about 10 °C [19]. In addition, constructing an additional disulfide bridge in TrxII (T2C-T28C) from *Trichoderma reesei* resulted in the half-life from ~ 40 s to ~ 20 min at 65 °C [20]. Thus, it has been proposed that improvement of heat resistance was affected by slight differences in the structural positions of disulfide bridges in the N-terminal region [19]. The stabilizing effect of the disulfide bridge in PjxA-DB (T2C-T29C) is remarkable. Structural analysis showed that the disulfide bridge T2C-T29C on PjxA-DB located between an irregular loop and a β-strand A2 (Fig. 5b), significantly increased its thermostability.

Usually thermophilic GH11 xylanases possess a β-strand in the N-terminal region, while mesophilic GH11 xylanases have a long irregular loop at the same position [30]. It is more likely that the reason PjxA was unstable at elevated temperature is the looseness of the irregular loop.

In contrast, the extra disulfide bridge seemed to attach the loop tightly to a protein core, instead of hanging freely as a flexible segment. Interestingly, the recombinant xylanases PjxA-DB and PTxA-DB formed a few dimers compared to the wild type (Fig. 2). It seems that the introduction of intramolecular disulfide bridge C2-C29 significantly improved the hydrophobicity, and the free Cys residue (C2, C29, C43) in the molecular surface was closer than the wild type, then the monomers tended aggregation to form a dimer. Previous studies have found intermolecular disulfide bridges have a positive effect on heat resistance [31]. In this study, the number of dimers was far less than monomers, which indicates that the intramolecular disulfide bridge C2-C29 in the N-terminal region is the key reason for the improvement of thermostability. Notably, the recombinant xylanases that have disulfide bridges also have increased specific activity and substrate affinity (Table 2). Recently, the N-terminal residues of *Aspergillus niger* xylanase (Xyn) were shown to have more impact on its catalytic efficiency and substrate-binding affinity compared to those on the C-terminus [32]. The catalytic characteristics of Srxyn from *Strepromyce rochei* L10904 were improved by modifications to the N-terminal region through site-directed mutagenesis in the cord [33]. However, the reason why the N-terminal disulfide bridges of PjxA-DB and PTxA-DB increased specific activity and substrate affinity is unclear. A possible explanation could be that the disulfide bridges in the N-terminal region greatly alter the nearby structures by modifying the putative substrate-binding sites, which has effected the catalysis process.

To our surprise, combining two mutation approaches produced the recombinant xylanase PTxA-DB with greater thermostability than by using either one alone (Figs. 3 and 4). Previous studies revealed hydrophobic interactions play an important role in stabilizing the three-dimensional structure of proteins [34, 35]. The hydrophobicity analysis revealed that the mutations of residues T2C, T29C, N30 L and Y15F might have improved the hydrophobicity of the N-terminal region in PTxA-DB. The formation of disulfide bridges requires strict stereo-chemical conformations and the position of residues [8]. When combined with N30 L in β-strand A2, Cys-29 is adjacent to the hydrophobic amino acid Leu30 and might delay the disulfide bridge (T2C-T29C) cleavage at high temperature. The introduction of cysteines tends to form the hydrophobic clusters in the N-terminal region [17]. Then, the van der Waals interactions can be enhanced, and the tighter packing can be achieved, the stabilization of N-terminal region has been greatly improved, especially the irregular loop where the xylanase unfolding was initiated. It seems that the effects of amino acid substitutions on thermostability are additive and cooperative. Consequently, the thermostability of PTxA-DB is higher than PjxA-DB, PTxA and the wild type PjxA.

## Conclusion

The thermostability of PjxA has been enhanced by constructed the disulfide bridge (T2C-T29C) between the irregular loop and the β-strand A2. It is indicated T2C, T29C, N30 L and Y15F lead to increase N-terminal hydrophobicity. The Tm value of mutant PTxA-DB was improved from 21.3 °C to 76.6 °C, and its half-life was found to be 53.6 min at 60 °C, 107-fold higher than the wild type strain. Moreover, the specific activity and substrate affinity of PTxA-DB were also enhanced under the acidic pH values. These results indicated that PTxA-DB could be a prospective additive to industrial animal feeds.

## Methods

### Strains, plasmids, culturing conditions

Acidic xylanase gene *pjxA* (GenBank accession code MK948222) was amplified from *Penicillium janthinellum* (MA21601), and the strain was screened by *Beijing Advanced Innovation Center for Food Nutrition and Human Health*. The whole sequence was about 720 bp in length with only one intron of 63 bp. The cDNA sequence was 657 bp long and putatively encoded a protein which contained a 28-amino acids signal peptide and a 190- amino acids mature peptide. Plasmid PET-28a (TIANGEN Bio-tek Co., Ltd., Beijing, China) and *Escherichia coli* BL21 (DE3) (TIANGEN Bio-tek Co., Ltd., Beijing, China) were used as the cloning vector host. The sequence of the excised signal peptide was cloned into the pET-28a vector. Restriction endonucleases, T4 DNA ligase and Q5® High-Fidelity DNA polymerase were obtained from NEB Ltd. (NEB Ltd., Ipswich, MA, USA). Beechwood xylan was obtained from Megazyme (P-XYLANBE-20G, Wicklow, Ireland). The cells were grown in LB medium, and isopropyl β-D-1-thiogalactopyranoside (IPTG) was used to induce the expression of the gene encoding xylanase under the condition of 20 °C, 200 r/min, and 16 h.

### Construction of recombinant xylanases

Five amino acids residues (T10Y, N11H, N12D, Y15F, N30 L) in N-terminus of PjxA to carry out substitutions with the thermophilic xylanase TfxA from *Thermomonospora fusca* [15, 16] was used to construct PTxA, based on sequence alignment and structural analysis. The mutation strategy was introduced to the original enzyme to form hydrophobic interactions. The second mutation strategy was introduced an additional disulfide bridge to construct PjxA-DB. Two residues of PjxA, threonine-2 and threonine-29, were identified as the target amino acids by analysis of a suitable position for a new disulfide bridge and then changed to cysteines using site-directed mutations. Adding a disulfide bridge to the N-terminus can improve the thermostability of recombinant xylanases in previous reports [21, 22, 36]. In this study, we combined both strategies, constructed PTxA-DB, to further enhance the thermostability.

### Protein expression and purification

Acidic xylanase gene *pjxA* was amplified from *Penicillium janthinellum* (MA21601) by genome walking. All genes of the mutants were cloned by overlap extension PCR. All mutated genes were digested with *NcoI* and *NotI* and ligated in PET-28a, then transformed into *E. coli* BL21 (DE3). The resulting recombinant xylanases were induced by addition of IPTG after sequence confirmation. Cells were harvested by centrifugation (8000×g, 10 min, 4 °C) and suspended in 10 mL 50 mM sodium acetate buffer (pH 5.5). The crude enzyme solution was obtained from the supernatants after ultrasonication. The recombinant xylanases were purified using Ni-column equilibrated in 50 mM phosphate buffer (pH 8.0) including 0.3 M NaCl and different concentrations of imidazole (0–0.2 M). The purity of the xylanases were assessed using SDS-PAGE method of Laemmli [24]. All protein concentrations were determined by the BCA method (BSA as the protein standard).

### Enzyme assays

Xylanase activity was evaluated by measuring the amount of reducing sugars released from beechwood xylan using the 3, 5-dinitrosalicylic acid (DNS) method with xylose as a standard [37].0.1 mL of diluted enzyme sample was mixed with 0.9 mL of substrate solution (1%) in 50 mM sodium acetate buffer (pH 5.5) and incubated for 5 min at 55 °C. Then 1 mL DNS solution was added and boiled for 15 min to terminate the reaction [38]. Reducing sugars (Calculated by xylose, xylose concentration for the standard curve are 4 to 20 μmol/ml) were determined from the increase in absorbance at 540 nm. One unit of xylanase activity was defined as the amount of enzyme that produced reducing sugars equivalent to 1 μmol of xylose per minute.

### Measurements of pH and temperature characteristics in recombinant xylanases

The effect of pH on the activity of xylanase was studied in two different buffers (0.1 M) at pH 2.0 to 6.5; Glycine hydrochloride buffer was used for the pH range 2.0 to 3.0; citrate buffer for pH 3.5 to 6.5. To determine the pH stability of the enzyme, the xylanase was incubated in the previously mentioned two different buffers in different pH range at 40 °C for 30 min and the residual xylanase activities were measured by the standard assay procedure.

The xylanase activity was examined from 45 to 75 °C to determine the optimum temperature using 1% beechwood xylan as a substrate in 50 mM sodium acetate buffer (pH 5.5) using DNS method as described above. The temperature at which xylanase has the highest activity is the temperature optimum. Thermostability of mutants were measured without substrate after 30 min of incubation at a temperature ranging between 45 and 65 °C. To determine whether the introduction of the disulfide bridge enhanced the heat resistance, the reconstructed xylanases PjxA, PjxA-DB and PTxA-DB were treated with 10 mM DTT at 4 °C for 12 h, the enzyme without treatment was used as the control [39]. The half-lives(t_1/2_) of recombinant xylanases were determined by incubating different time at 60 °C, t_1/2_ was calculated following the equation y = A*e^-kt^, where A is the equation preferencet is the inactivation time and k is the slope. It was fitted with the residual activity-time curves and the t_1/2_ = ln2/k [12].

### Differential scanning calorimetry (DSC)

ThePjxA and mutated xylanases were diluted to a final concentration of 0.5 mg/mL with 50 mM pH 5.5 acetate buffer. The thermal stability of the enzymes was examined using Nano-DSC at a scanning speed of 1 °C /min. The heating process system ranged from 25 to 85 °C, and was equilibrated before each sample measurement [17, 40]. A graph was generated by the Launch Nano Analyze software which generated the denaturation temperature (*T*_m_).

### Enzymes kinetic parameters

The reaction duration was set at 5 min with a concentration of beechwood xylan ranging from 0.2 to 2.4% (w/v) to determine the kinetic parameters. Xylanase activity was examined in 50 mM citrate buffer (pH 4.5) at 50 °C, using the DNS method to estimate reducing sugars. The *K*_m_ and *k*_cat_ values of PjxA and other recombinant xylanases were calculated according to the *GraphPad Prism software*.

## Additional file

Additional file 1: **Table S1.** The thermostability effect of a disulfide bridge. **Table S2.** The thermostability of xylanases. **Figure S1.** The original picture of SDS-PAGE (SDS-PAGE in Fig. 2). **Figure S2** LC-MS spectra of PjxA-DB (monomer). **Figure S3.** LC-MS spectra of PTxA-DB (monomer). **Figure S4.** LC-MS spectra of PjxA-DB-2 (dimer). **Figure S5.** LC-MS spectra of PTxA-DB-2 (dimer). **Figure S6.** The Michaelis-Menten plots of recombinant xylanases. Primers and sequences of recombined xylanases. (DOC 810 kb)

## Data Availability

The data collected upon which this article is based upon are all included in this manuscript and the Additional file associated with it.

## Abbreviations

DNS: 3, 5-dinitrosalicylic acid
DSC: Differential Scanning Calorimetry
IPTG: Isopropyl β-D-1-thiogalactopyranoside
Tm: Denaturation temperature
