# Supplementary material for: Mutagenesis of N-terminal residues confer thermostability on a Penicillium janthinellum MA21601 xylanase
Source: BMC Biotechnol. 2019 Jul 25;19:51. doi: 10.1186/s12896-019-0541-7 (PMC6659274; doi:10.1186/s12896-019-0541-7)
Supplement: Supplementary file 1 — Table S1. The thermostability effect of a disulfide bridge. Table S2. The thermostability of xylanases. Figure S1. The original picture of SDS-PAGE (SDS-PAGE in Fig. 2). Figure S2 LC-MS spectra of PjxA-DB (monomer). Figure S3. LC-MS spectra of PTxA-DB (monomer). Figure S4. LC-MS spectra of PjxA-DB-2 (dimer). Figure S5. LC-MS spectra of PTxA-DB-2 (dimer). Figure S6. The Michaelis-Menten plots of recombinant xylanases. Primers and sequences of recombined xylanases. (DOC 810 kb) [file 12896_2019_541_MOESM1_ESM.doc]

**Supplemental data**

**Table. S1** The thermostability effect of a disulfide bridge.

| Xylanases | PjxA | | PjxA-DB | | PTxA-DB | |
| --- | --- | --- | --- | --- | --- | --- |
| DTT | - | + | - | + | - | + |
| Residual activity（%） | 5.18 | 4.47 | 83.56 | 5.74 | 82.09 | 12.33 |

Samples were treated with 10 mM DTT at 4 ^o^C for 12 h, the xylanases without treatment were used as the control. Residual activities of xylanases were determined by incubating 30min at 50℃. All enzyme concentrations were adjusted to 0.05 mg/mL.

**Table. S2** The thermostability of xylanases.

| Xylanases | Mutations | | Residual activity  (%) | | | Optimal temperature  (^o^C) |
| --- | --- | --- | --- | --- | --- | --- |
| PjxA | | --- | | 3.6 | 50 | |
| PTxA-4 | | T10Y/N11H/N12D/Y15F | | 8.6 | 50 | |
| PTxA-1 | | N30L | | 3.8 | 50 | |
| PTxA | | T10Y/N11H/N12D/Y15F/N30L | | 14.5 | 50 | |

The mutation of N-terminal crucial region and amino acid in xylanase could synergistically improve its thermostability. Thermostability of mutants were measured without substrate after 30 min of incubation at 50℃. All enzyme concentrations were adjusted to 0.05 mg/mL. PjxA: recombinant xylanase of *Penicillium janthinellum* (MA21601); PTxA-4: substitution of N-terminal crucial region (T10Y/N11H/N12D/Y15F); PTxA-1: mutation of N-terminal amino acid N30L; PTxA: substitution of N-terminal crucial region (T10Y/N11H/N12D/Y15F) and amino acid (N30L).


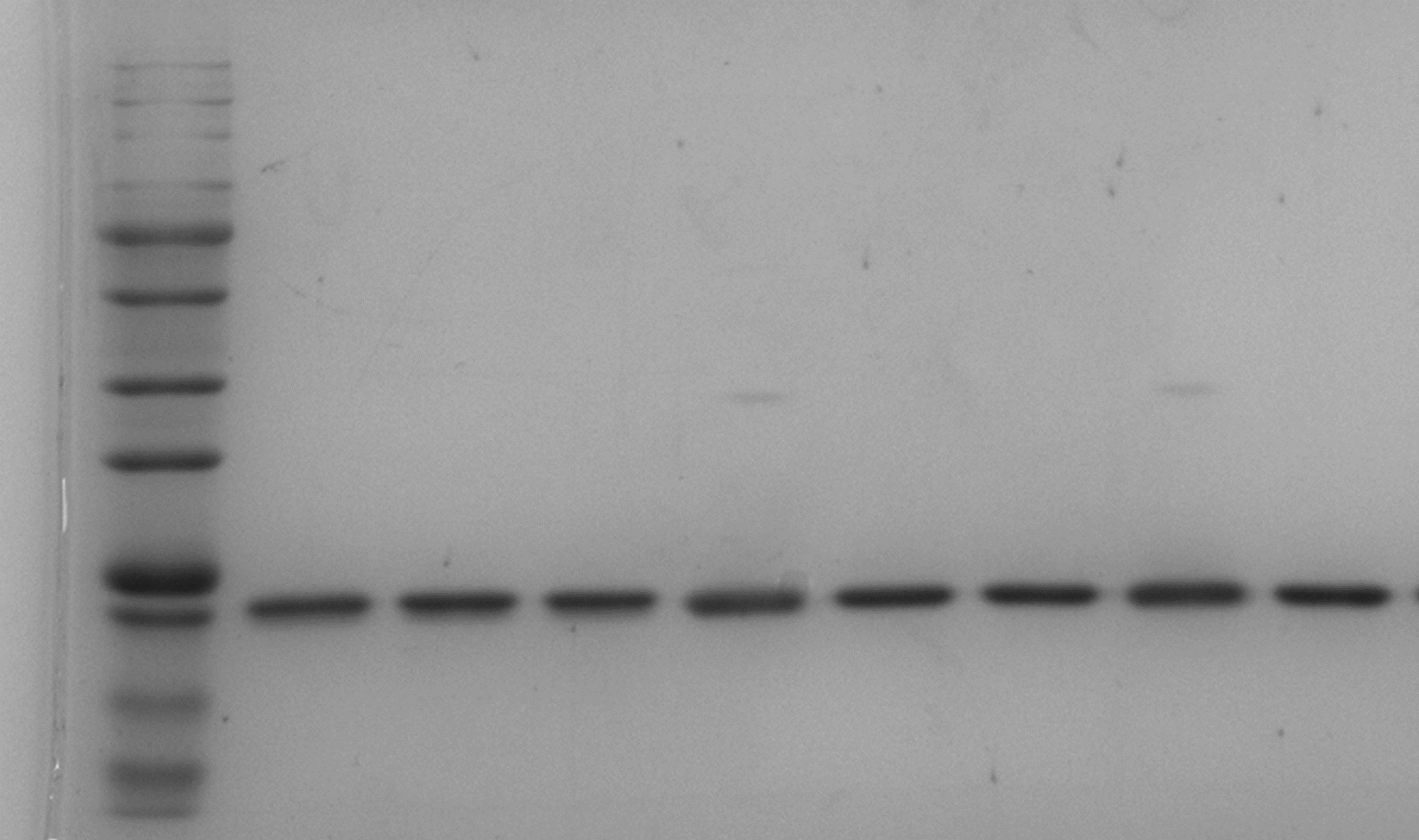


**M 1 2 3 4 5 6 7 8**

**+ - + - + - + DDT**


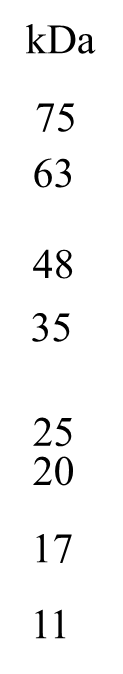


**Figure S1 The original picture of SDS-PAGE (SDS-PAGE in figure 2).**

Lane M, the molecular weight marker; lane 1, PjxA(-DTT); lane 2, PjxA(+DTT); lane 3, PTxA(-DTT); lane 4, PTxA(+DTT); lane 5, PjxA-DB(-DTT); lane 6, PjxA-DB(+DTT); lane 7, PTxA-DB(-DTT); lane 8, PTxA-DB(+DTT).


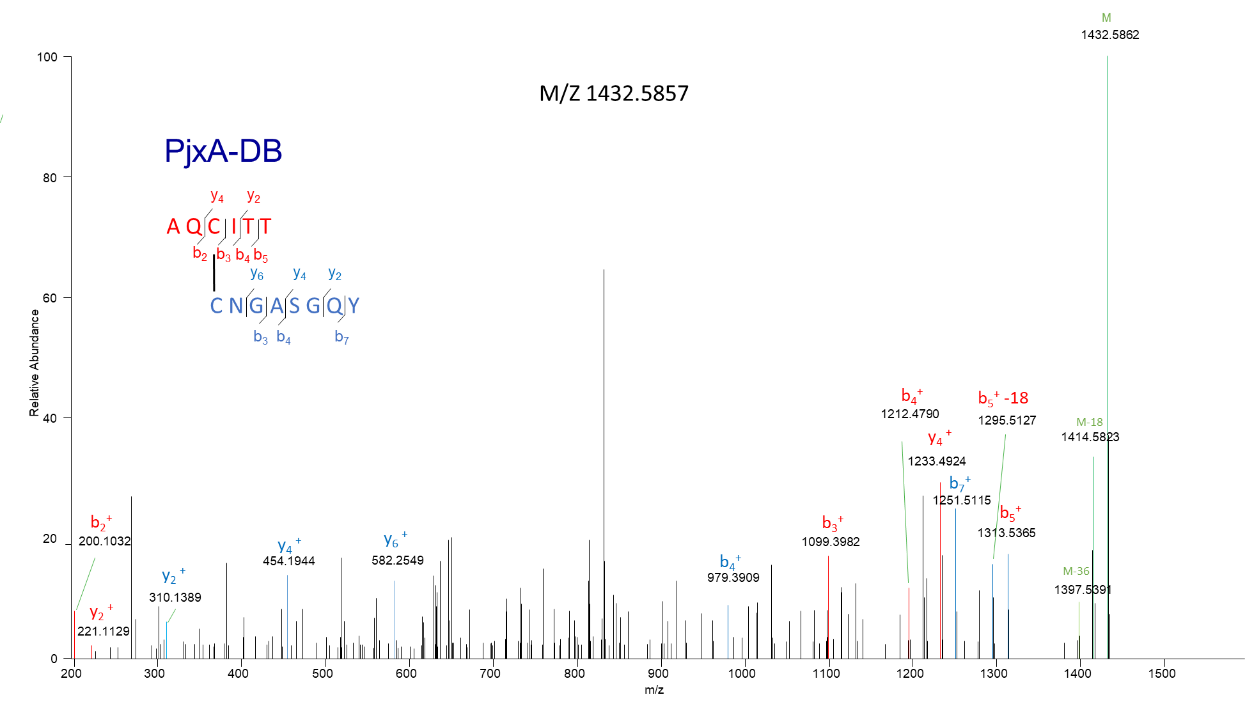


**Figure. S2** LC-MS spectra of PjxA-DB (monomer).


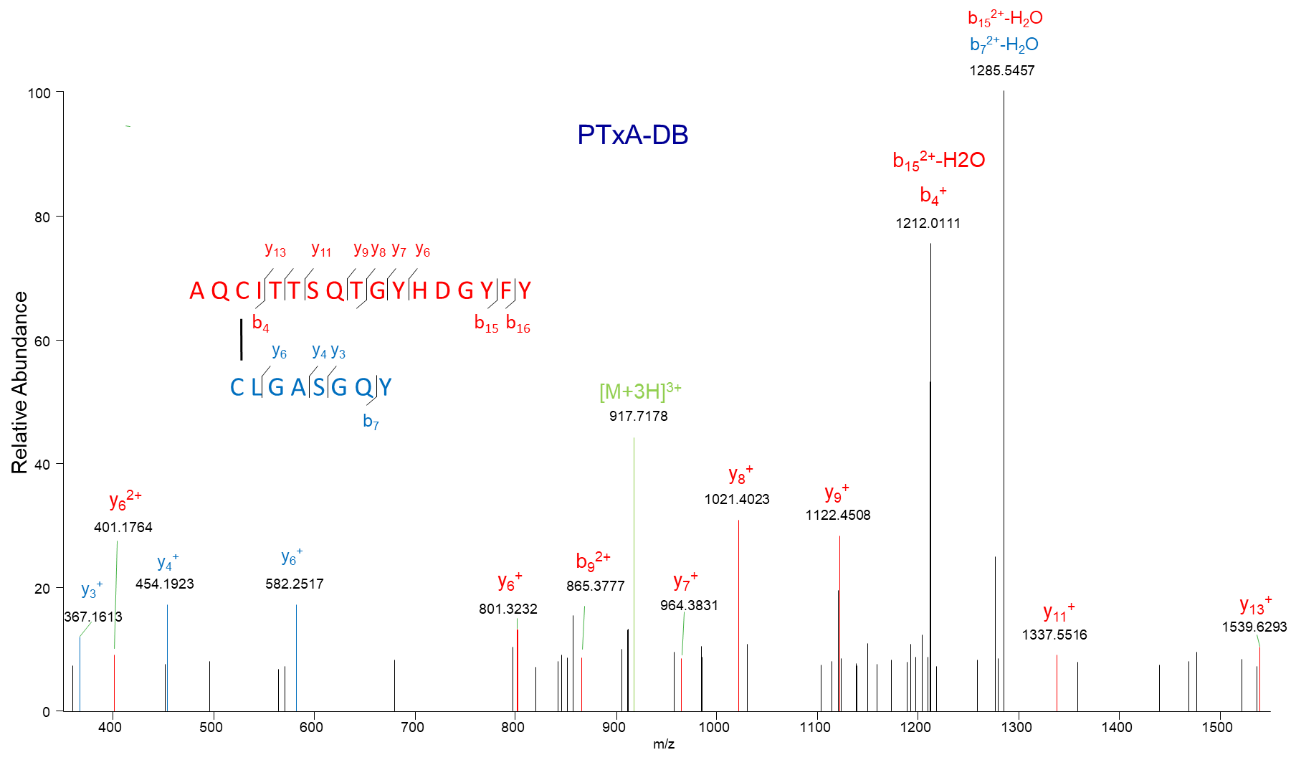


**Figure. S3** LC-MS spectra of PTxA-DB (monomer).


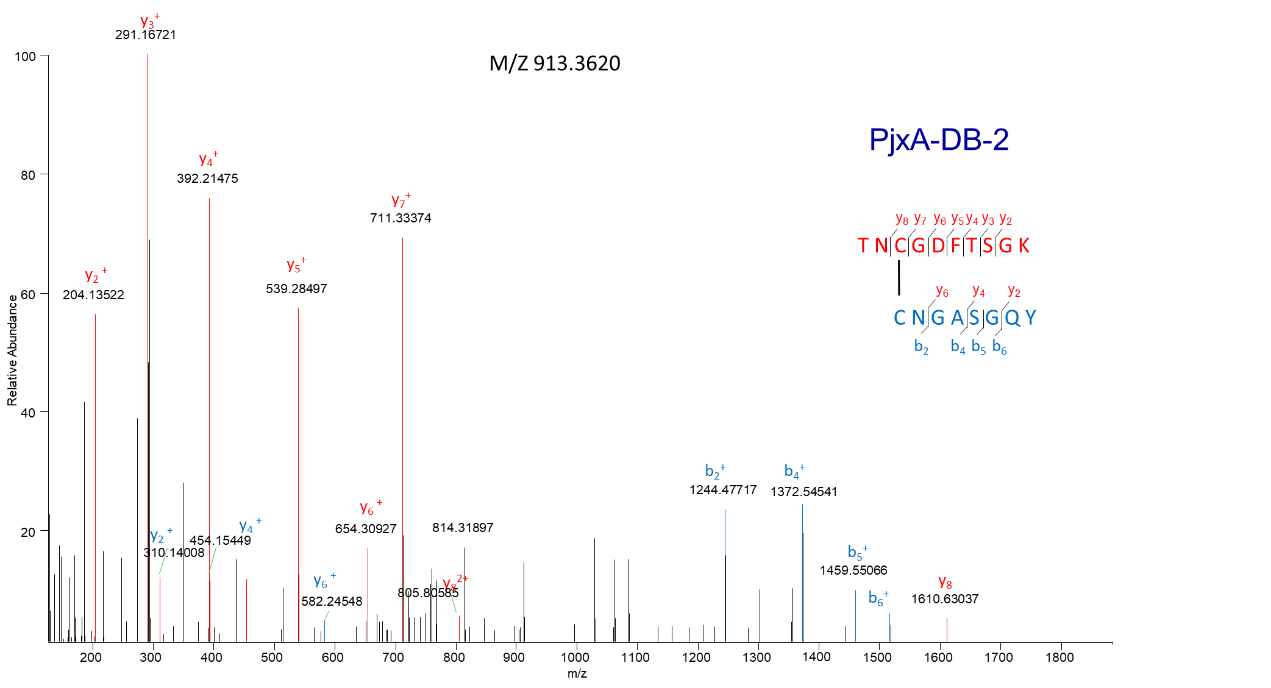


**Figure. S4** LC-MS spectra of PjxA-DB-2 (dimer).


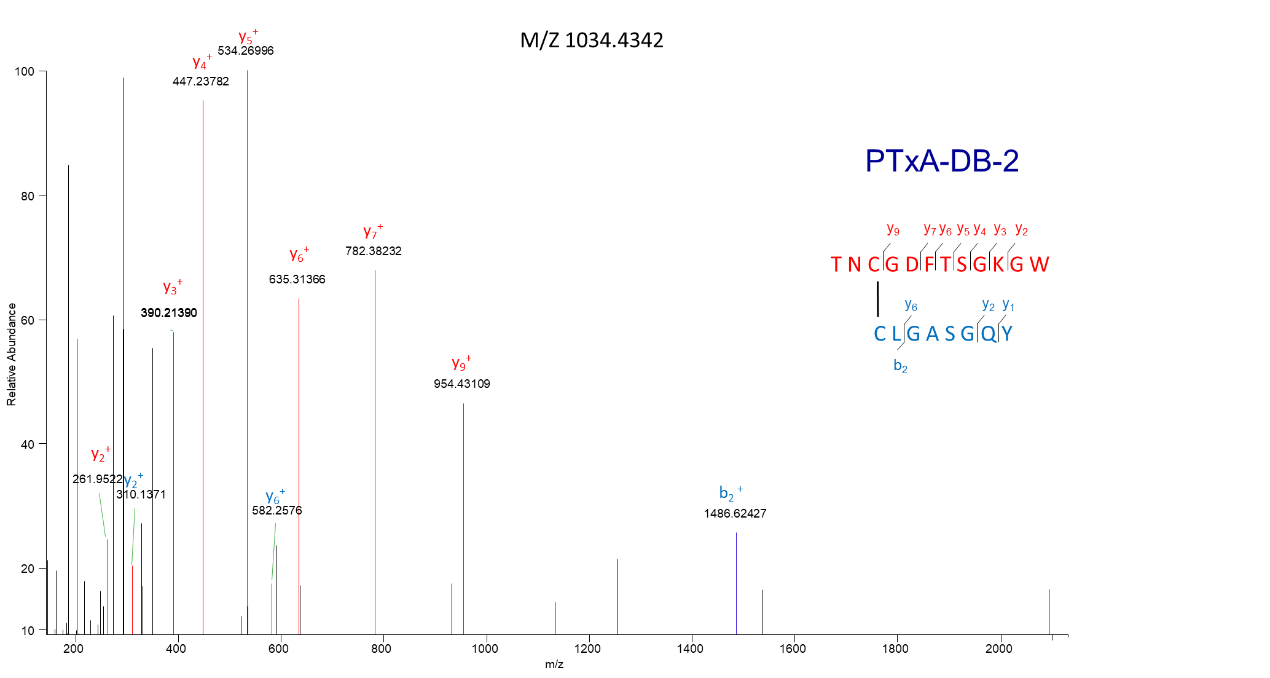


**Figure. S5** LC-MS spectra of PTxA-DB-2 (dimer).

There are intramolecular disulfide bonds 2-29 in PjxA-DB and PTxA-DB (The first amino acid Ala in the figure, because the protected base in N-terminal cleavage site, it also existed in PjxA and did not affect the protein expression). We speculated that there might be a few non-specific intermolecular disulfide bonds between free C2, C29, and C43 residues. Here, the mass spectrum of the intermolecular disulfide bond C29-C43 of PjxA-DB2 (Figure.S3) and PTxA-DB2 (Figure.S4)was taken as an example.

**
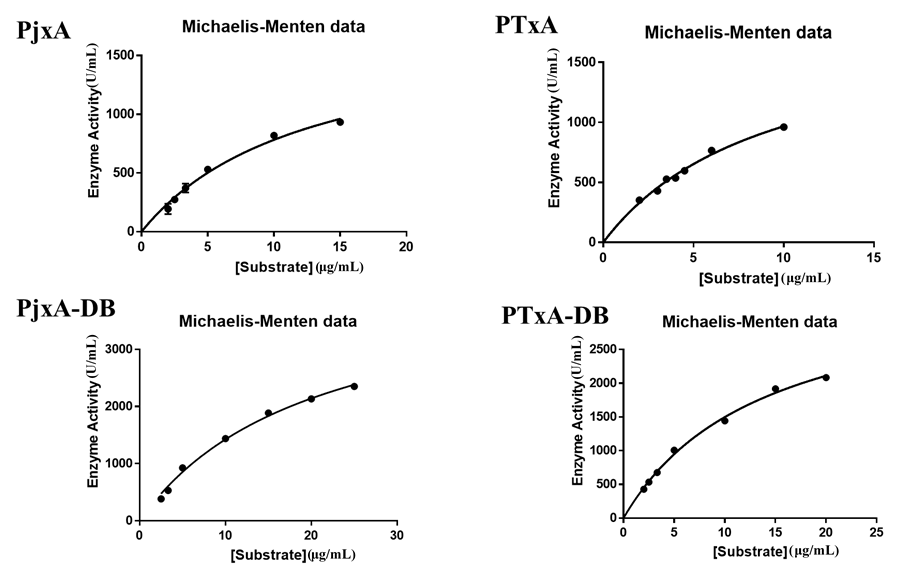
**

**Figure. S6** The Michaelis-Menten plots of recombinant xylanases.

The substrate concentrations were 0.2% to 1.5% (w/v) for PjxA, 0.2% to 1.0% (w/v) for PTxA, 0.2% to 2.5% (w/v) for PjxA-DB and 0.2% to 2.0% (w/v) for PTxA-DB. The concentration of each enzyme was adjusted to 0.05 mg/mL and dialyzed against their optimal pH, the enzyme assay was under their optimal temperatures.

**Primers and sequences of recombined xylanases**

1. **PjxA** (wild type)

Primers:

PjxA-F: 5’-CATGCCATGGCACAGACCATTACCACCAGCCAGACT-3’ Tm 64.3

PjxA-R: 5’-TTGCGGCCGCCGAAACAGTGATGGAAGAAGAACCA-3’ Tm 64.6

Gene sequence:

CAGACCATTACCACCAGCCAGACTGGAACCAACAATGGCTACTACTACTCCTTCTGGACCAACGGTGGTGGTACCGTCAGCTACACCAATGGTGCTAGCGGACAGT ACAGCGTCTC CTGGACGAACTGCGGTGACTTCACCTCCGGCAAGGGTTGGGCCACTGGAAGCGCCCGGAACATCAACTTCTCGGGCTCCTTCAACCCCTCGGGTAACGCCTACCTTGCTGTTTATGGCTGGACTACTAGCCCCCTCGTCGAGTACTACATCCTCGAGAACTACGGTACCTACAACCCGGGCTCCAGCATGACCCACAAGGGTACCGTCTACAGCGATGGCGCTACCTACGACATCTACGA GCACCAGCAA GTCAACCAGC CCTCCATCCA AGGCACTGCGACCTTCAACC AGTACTGGTCCATCCGCCAGAGCAAGCGTTCCAGCGGCACTGTGACCACTGCCAACCACTTCAATGCTTGGGCCAAGTTGGGAATGAACCTGGGTAGCTTCAACTACCAGATCGTTTCCACTGAGGGTTACCAGAGCAGTGGTTCTTCTTCCATCACTGTTTCGTAA

Protein sequence:

QTITTSQTGTNNGYYYSFWTNGGGTVSYTNGASGQYSVSWTNCGDFTSGKGWATGSARNINFSGSFNPSGNAYLAVYGWTTSPLVEYYILENYGTYNPGSSMTHKGTVYSDGATYDIYEHQQVNQPSIQGTATFNQYWSIRQSKRSSGTVTTANHFNAWAKLGMNLGSFNYQIVSTEGYQSSGSSSITVS

1. **PTxA**

Template: PjxA

Primers:

PTxA F:

5’-CATGCCATGGCACAGACCATTACCACCAGCCAGACTGGATATCATGATGGCTATTTTTACTCCTTCTGGACCAACGGTGGT-3-3’ Tm=65.9

N30L-r 5’-3’ GCTAGCACCCAGGGTGTAGCTG 63.7

N30L-f 5’-3’ CAGCTACACCCTGGGTGCTAGC 63.7

PTxA-R: 5’-TTGCGGCCGCCGAAACAGTGATGGAAGAAGAACCA-3’ Tm 64.6

Gene sequence:

CAGACCATTACCACCAGCCAGACTGGATATCATGATGGCTATTTTTACTCCTTCTGGACCAACGGT GGTGGTACCG TCAGCTACAC CCTGGGTGCTAGCGGACAGT ACAGCGTCTC CTGGACGAACTGCGGTGACTTCACCTCCGGCAAGGGTTGGGCCACTGGAAGCGCCCGGAACATCAACTTCTCGGGCTCCTTCAACCCCTCGGGTAACGCCTACCTTGCTGTTTATGGCTGGACTACTAGCCCCCTCGTCGAGTACTACATCCTCGAGAACTACGGTACCTACAACCCGGGCTCCAGCATGACCCACAAGGGTACCGTCTACAGCGATGGCGCTACCTACGACATCTACGA GCACCAGCAA GTCAACCAGC CCTCCATCCA AGGCACTGCGACCTTCAACC AGTACTGGTCCATCCGCCAGAGCAAGCGTTCCAGCGGCACTGTGACCACTGCCAACCACTTCAATGCTTGGGCCAAGTTGGGAATGAACCTGGGTAGCTTCAACTACCAGATCGTTTCCACTGAGGGTTACCAGAGCAGTGGTTCTTCTTCCATCACTGTTTCGTAA

Protein sequence:

QTITTSQTGYHDGYFYSFWTNGGGTVSYTLGASGQYSVSWTNCGDFTSGKGWATGSARNINFSGSFNPSGNAYLAVYGWTTSPLVEYYILENYGTYNPGSSMTHKGTVYSDGATYDIYEHQQVNQPSIQGTATFNQYWSIRQSKRSSGTVTTANHFNAWAKLGMNLGSFNYQIVSTEGYQSSGSSSITVS

1. **PjxA-DB**

Template: PjxA

Primers:

PjxA-DB**-**F: 5’-CATGCCATGGCA CAGTGCATTACCACCAGCCAGACT 65.3

DS2-29-r: 5-‘TAGCACCATTGCAGTAGCTGACGG-3’ 66.4

DS2-29-f: 5’-CCGTCAGCTACTGCCTGGGTGCTA-3’ 66.4

PjxA-DB-R: 5’-TTGCGGCCGCCGAAACAGTGATGGAAGAAGAACCA-3’ Tm 64.6

Gene sequence:

CAGTGCATTACCACCAGCCAGACTGGAACCAACAATGGCTACTACTACTCCTTCTGGACCAACGGTGGTGGTACCGTCAGCTACTGCAATGGTGCTAGCGGACAGTACAGCGTCTCCTGGACGAACTGCGGTGACTTCACCTCCGGCAAGGGTTGGGCCACTGGAAGCGCCCGGAACATCAACTTCTCGGGCTCCTTCAACCCCTCGGGTAACGCCTACCTTGCTGTTTATGGCTGGACTACTAGCCCCCTCGTCGAGTACTACATCCTCGAGAACTACGGTACCTACAACCCGGGCTCCAGCATGACCCACAAGGGTACCGTCTACAGCGATGGCGCTACCTACGACATCTACGAGCACCAGCAAGTCAACCAGCCCTCCATCCAAGGCACTGCGACCTTCAACCAGTACTGGTCCATCCGCCAGAGCAAGCGTTCCAGCGGCACTGTGACCACTGCCAACCACTTCAATGCTTGGGCCAAGTTGGGAATGAACCTGGGTAGCTTCAACTACCAGATCGTTTCCACTGAGGGTTACCAGAGCAGTGGTTCTTCTT CCATCACTGT TTCGTAA

Protein sequence: QCITTSQTGTNNGYYYSFWTNGGGTVSYCNGASGQYSVSWTNCGDFTSGKGWATGSARNINFSGSFNPSGNAYLAVYGWTTSPLVEYYILENYGTYNPGSSMTHKGTVYSDGATYDIYEHQQVNQPSIQGTATFNQYWSIRQSKRSSGTVTTANHFNAWAKLGMNLGSFNYQIVSTEGYQSSGSSSITVS

**4. PTxA-DB**

Template: PTxA

Primers:

PTxA-DB-F: 5’-CATGCCATGGCA CAGTGCATTACCACCAGCCAGACT Tm 65.3

PTxA-DB r: 5-‘TAGCACCCAGGCAGTAGCTGACGG-3’ Tm 69.5

PTxA-DB f: 5’-CCGTCAGCTACTGCCTGGGTGCTA-3’ Tm 69.5

PTxA-DB-R: 5’-TTGCGGCCGCCGAAACAGTGATGGAAGAAGAACCA -3’ Tm 64.6

Gene sequence:

CAGTGCATTACCACCAGCCAGACTGGATATCATGATGGCTATTTTTACTCCTTCTGGACCAACGGTGGTGGTACCGTCAGCTACTGCCTGGGTGCTAGCGGACAGTACAGCGTCTCCTGGACGAACTGCGGTGACTTCACCTCCGGCAAGGGTTGGGCCACTGGAAGCGCCCGGAACATCAACTTCTCGGGCTCCTTCAACCCCTCGGGTAACGCCTACCTTGCTGTTTATGGCTGGACTACTAGCCCCCTCGTCGAGTACTACATCCTCGAGAACTACGGTACCTACAACCCGGGCTCCAGCATGACCCACAAGGGTACCGTCTACAGCGATGGCGCTACCTACGACATCTACGAGCACCAGCAAGTCAACCAGCCCTCCATCCAAGGCACTGCGACCTTCAACCAGTACTGGTCCATCCGCCAGAGCAAGCGTTCCAGCGGCACTGTGACCACTGCCAACCACTTCAATGCTTGGGCCAAGTTGGGAATGAACCTGGGTAGCTTCAACTACCAGATCGTTTCCACTGAGGGTTACCAGAGCAGTGGTTCTTCTTCCATCACTGT TTCGTAA

Protein sequence:

QCITTSQTGYHDGYFYSFWTNGGGTVSYCLGASGQYSVSWTNCGDFTSGKGWATGSARNINFSGSFNPSGNAYLAVYGWTTSPLVEYYILENYGTYNPGSSMTHKGTVYSDGATYDIYEHQQVNQPSIQGTATFNQYWSIRQSKRSSGTVTTANHFNAWAKLGMNLGSFNYQIVSTEGYQSSGSSSITVS
